# Connectivity across the Caribbean Sea: DNA Barcoding and Morphology Unite an Enigmatic Fish Larva from the Florida Straits with a New Species of Sea Bass from Deep Reefs off Curaçao

**DOI:** 10.1371/journal.pone.0097661

**Published:** 2014-05-13

**Authors:** Carole C. Baldwin, G. David Johnson

**Affiliations:** Department of Vertebrate Zoology, National Museum of Natural History, Smithsonian Institution, Washington D. C., United States of America; University of Guelph, Canada

## Abstract

Integrative taxonomy, in which multiple disciplines are combined to address questions related to biological species diversity, is a valuable tool for identifying pelagic marine fish larvae and recognizing the existence of new fish species. Here we combine data from DNA barcoding, comparative morphology, and analysis of color patterns to identify an unusual fish larva from the Florida Straits and demonstrate that it is the pelagic larval phase of a previously undescribed species of *Liopropoma* sea bass from deep reefs off Curaçao, southern Caribbean. The larva is unique among larvae of the teleost family Serranidae, Tribe Liopropomini, in having seven elongate dorsal-fin spines. Adults of the new species are similar to the golden bass, *Liopropoma aberrans,* with which they have been confused, but they are distinct genetically and morphologically. The new species differs from all other western Atlantic liopropomins in having IX, 11 dorsal-fin rays and in having a unique color pattern–most notably the predominance of yellow pigment on the dorsal portion of the trunk, a pale to white body ventrally, and yellow spots scattered across both the dorsal and ventral portions of the trunk. Exploration of deep reefs to 300 m using a manned submersible off Curaçao is resulting in the discovery of numerous new fish species, improving our genetic databases, and greatly enhancing our understanding of deep-reef fish diversity in the southern Caribbean. *Oh the mother and child reunion is only a moment away*. **Paul Simon**.

## Introduction

New marine fish species are described at an average rate of 100–150 per year, with recent studies estimating an additional 3,300–5,000 yet to be described for a total of 19,000–22,000 [1], [2]. Deep reefs and deep slopes, which are below depths accessible by conventional scuba gear, represent ecosystems where most new marine fishes are likely to be found [1]. New fish species have traditionally been recognized through comparative morphological analyses, but DNA barcoding [3], which involves sequencing approximately 650 base pairs of the mitochondrial gene cytochrome c oxidase subunit I (COI), has proved useful in identifying numerous cryptic new fish species [4], [5], even in well-studied shallow regions of the ocean such as the Caribbean Sea [6]–[10]. DNA barcoding also is demonstrably valuable in linking marine-fish larvae to adults [11]–[20], a historically difficult task at the species level because larvae often bear little morphological resemblance to adults [21], [22]. Integrative taxonomy, in which disciplines such as comparative morphological and molecular analyses are combined to address questions related to biological species diversity, is a promising tool for identifying pelagic marine fish larvae and recognizing the existence of new fish species [5]–[10], [17], [18]. Nevertheless, species identifications of larvae of most marine fishes remain elusive.

**Figure 1:**
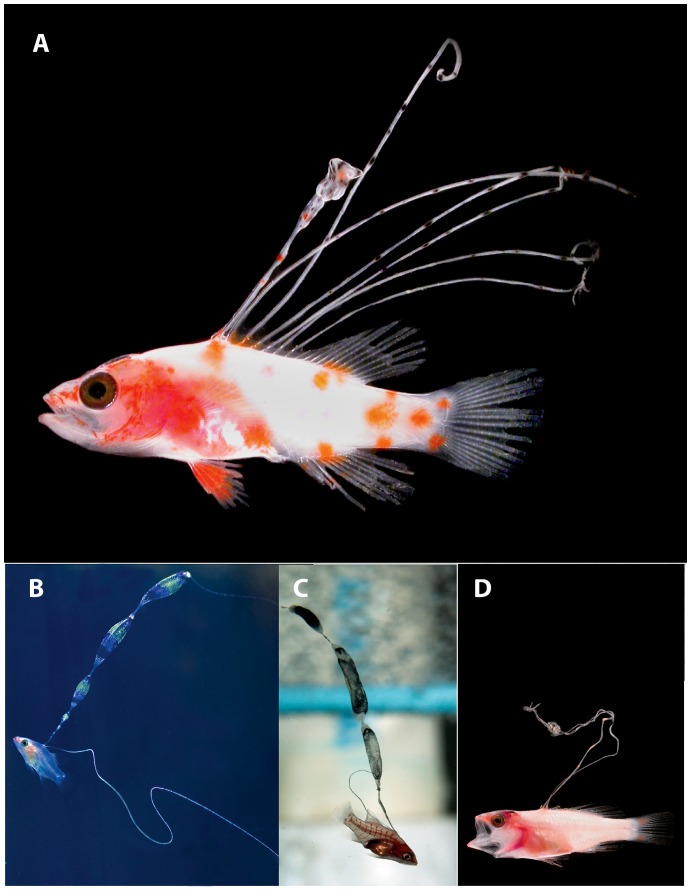
Larvae of western Atlantic Liopropomini. A, *Liopropoma olneyi* sp. nov, USNM 426868, paratype, DNA #FLST 5001, 14.0 mm SL, Florida Straits, photo by Cedric Guigand. B, *L. rubre,* reared aquarium larva, photo by Christopher Paparo. C, *L. carmabi,* reared aquarium larva, photo by Christopher Paparo; D. *Bathyanthias* sp., Florida Straits, photo by Cedric Guigand.

In a paper on marine population connectivity [23], several color photographs of fresh specimens of larval fishes were included without legends or identifications. One of the most striking of these [23] was a perciform larva with seven very elongate dorsal-fin spines (Fig. 1A). We were intrigued by the larva because it most closely resembles larvae of the epinepheline serranid tribe Liopropomini, which typically have only two elongate dorsal-fin spines (Fig. 1B–D–see also [19], [24], [25]). The only known epinepheline larva with numerous elongate dorsal-fin spines is that of *Belonoperca chabanaudi* [25], a member of the exclusively Indo-Pacific tribe Diploprionini [26]. Our initial surmise that the unknown larva had been collected in the Indo-Pacific proved wrong, as Robert Cowen (University of Miami/Oregon State University, pers. com.) and Cedric Guigand (University of Miami, pers. com.) confirmed that, like the other specimens figured in the paper, it was collected in the Florida Straits. We now have the specimen in hand, which was preserved in 95% ethanol, and after removing the right eye for genetic analysis and taking radiographs and additional photographs, we cleared and stained it. We confirmed that it is a liopropomin serranid [26], [27], but could not match it genetically or morphologically to any known liopropomin species. Morphologically it is unique among the tribe in having nine dorsal-fin spines and 11 dorsal soft rays (IX, 11). A second larval specimen from the Gulf of Mexico was collected by Tracey Sutton (Virginia Institute of Marine Science/NOVA Southeastern University), and it has the same unusual dorsal-fin count.

While we were working on the description of a new species based on the two larval specimens, one of the authors (CCB) and Ross Robertson (Smithsonian Tropical Research Institute) were exploring the deep-reef fish fauna off Curaçao in the southern Caribbean. This work, which is part of the Smithsonian Institution’s Deep Reef Observation Project (DROP), involves submersible diving to 300 m and is resulting in the discovery of numerous new species [28], [29]. Among the deep-reef fish specimens collected were multiple “golden basses,” which were identified as *Liopropoma aberrans* (Poey, 1860) based on general color pattern [30]. Genetic analyses of those specimens, however, revealed more than one lineage of *L. aberrans*, and morphological examination of the specimens revealed that one of the lineages is characterized by IX, 11 dorsal-fin rays. Combining available DNA barcoding data for western Atlantic liopropomin specimens yielded an unexpected discovery: the liopropomin larva from the Florida Straits is the pelagic stage of a cryptic new species of *Liopropoma* from southern Caribbean deep reefs. Here we present the genetic and morphological evidence, describe the new species, comment on the status of *L. aberrans,* and note the need for additional investigation of deep reefs–diverse but underexplored tropical ocean ecosystems worldwide.

## Materials and Methods

Deep-reef fish specimens were collected using the *Curasub* submersible (Substation Curaçao - http://www.substation-Curacao.com). The fish anesthetic quinaldine was pumped from a reservoir through a tube attached to one hydraulic arm, and specimens were collected with a suction hose attached to another arm. The latter empties into a vented plexiglass cylinder attached to the outside of the sub. Field protocol for processing specimens involved taking digital color photographs of fresh color patterns using a Fuji Finepix S3 or Nikon D600 digital camera and subsequently taking a muscle-tissue sample for genetic analysis. Voucher specimens were then preserved and archived at the Smithsonian’s National Museum of Natural History. Institutional abbreviations are as listed at http://www.asih.org/node/204.

Methods for making counts and measurements of larvae and adults and method of clearing and staining followed those of previous authors [30]–[34]. Measurements of adults were made with needle-point dial calipers or from images created using a Zeiss Axiocam in a Zeiss Discovery V12 dissecting microscope; measurements of larvae were made using the latter. Images of preserved and cleared-and-stained larvae were taken with the Zeiss Axiocam attached to a Zeiss Stereo Discovery V12. These pictures are composite images prepared with the Zeiss AxioVision software to increase the depth of field.

Tissue samples for DNA Barcoding were stored in saturated salt-DMSO (dimethyl sulfoxide) buffer [35]. DNA extraction, PCR, sequencing cytochrome c oxidase subunit I (COI), and editing COI sequences were performed as outlined by Weigt et al. [36]. A neighbor-joining tree for visualization purposes [37] was generated using PAUP*4.1 [38] on an analysis of Kimura two-parameter distances [39]. The neighbor-joining tree shows genetic distances in COI among individuals and how they cluster into genetically distinct lineages. Interspecific phylogenetic relationships were hypothesized for western Atlantic liopropomins based on maximum parsimony analysis of the COI sequences using heuristic searches in PAUP*4.1. Characters were equally weighted and left unordered. The resulting equally parsimonious trees were summarized using the strict consensus method, and nodal support was estimated from 1,000 replicates of the bootstrap, utilizing random addition sequence and TBR branch swapping [38]. Outgroups for both analyses were two members of the sister group of the Liopropomini–*Grammistes sexlineatus* and *Rypticus carpenteri* of the tribe Grammistini [27], and the trees were rooted on a more distant outgroup, *Scorpaena plumieri* of the family Scorpaenidae. The label for each entry on the neighbor-joining tree is our DNA number, and we include that number in the designation of type specimens and in some figure captions. Abbreviations used in DNA numbers are as follows: BAH – Bahamas, BLZ – Belize, CUR–Curacao, FLST–Florida Straits, FWRI – Florida Wildlife Research Institute, MBIO – Moorea Biocode Project, MCgroup – Matthew Craig, MOC – *Miguel Oliver* Caribbean Cruise, TOB - Tobago. GenSeq nomenclature for DNA sequences [40] and GenBank information are presented along with museum catalog numbers for voucher specimens in Appendix S1.

### Ethics Statement

Specimens were collected under the auspices of the Curaçao Sea Aquarium with permission of owner Adriaan Schrier. This study was carried out under Smithsonian Animal Care and Use Committee (ACUC) approval to C. C. Baldwin (ACUC #2011-07). *Guidelines for field activities with wild fishes* established by the American Society of Ichthyologists and Herpetologists (http://www.asih.org/files/fish%20guidelines.doc) were followed for all field collecting activities, including euthanasia with tricaine methane sulfate (MS-222). The field studies involved no endangered or protected species.

#### Nomenclatural acts

The electronic edition of this article conforms to the requirements of the amended International Code of Zoological Nomenclature, and hence the new name contained herein is available under that Code from the electronic edition of this article. This published work and the nomenclatural acts it contains have been registered in ZooBank, the online registration system for the ICZN. The ZooBank LSIDs (Life Science Identifiers) can be resolved and the associated information viewed through any standard web browser by appending the LSID to the prefix “http://zoobank.org/”. The LSID for this publication is: urn:lsid:zoobank.org:pub:23209F5C-7FFF-4E3D-ACDE-9A03E90025C2. The electronic edition of this work was published in a journal with an ISSN, and has been archived and is available from the following digital repositories: PubMed Central, LOCKSS.

## Results

**Figure 2:**
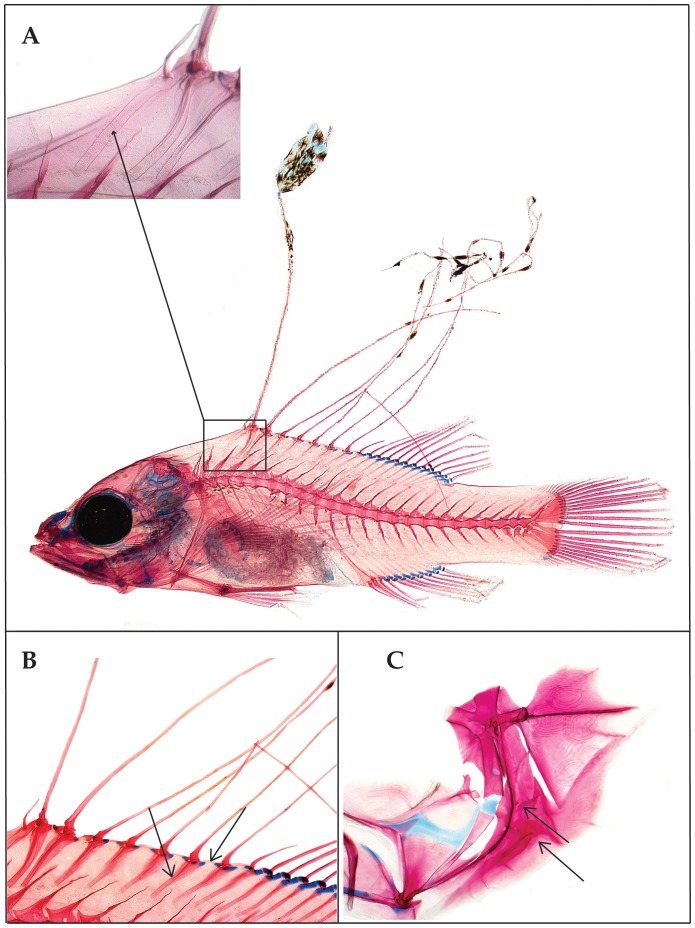
Photomicrographs of cleared-and-stained larval *Liopropoma olneyi,* sp. nov. (USNM 426868, paratype, 14.0 mm SL) showing morphological evidence of its placement in the epinepheline serranid tribe Liopropomini. A, whole specimen showing elongate, filamentous dorsal-fin spines, and inset showing close-up of thin, curved, first dorsal-fin pterygiophore. B, close-up of the spinous dorsal fin and fin supports showing separation of the distal radials of the third through last spinous dorsal-fin pterygiophores from the serially associated proximal-middle radials (right black arrow), and presence of a pterygiophore in the 7^th^ interneural space (left black arrow). C, close-up of bones of the opercular series showing absence of an elongate spine at the angle of the preopercle (lower black arrow) and presence of tiny spines on the lateral preopercular margin (upper black arrow). Photos by G. David Johnson.

Baldwin & Johnson [27] diagnosed five epinepheline serranid tribes and hypothesized relationships among them based on derived morphological features of both larvae and adults. The presence in the Florida Straits larva of the following combination of characters provides evidence that it is a member of the epinepheline tribe Liopropomini (the character number from [27] is given parenthetically for each character): the elongate anterior dorsal-fin spines are filamentous (Figs. 1A, 2A, character 14); there is no elongate spine at the angle of preopercle (Fig. 2C, character 15); the first dorsal-fin pterygiophore is thin and curved (Figs. 2A, character 20); the distal radials of the third through last spinous dorsal-fin pterygiophores are separated from the serially associated proximal-middle radials (Fig. 2B, character 21); a pterygiophore is present in the 7^th^ interneural space (Fig. 2B, character 29); and tiny spines are present on the lateral margin of preopercle (Fig. 2C, character 35).

**Figure 3:**
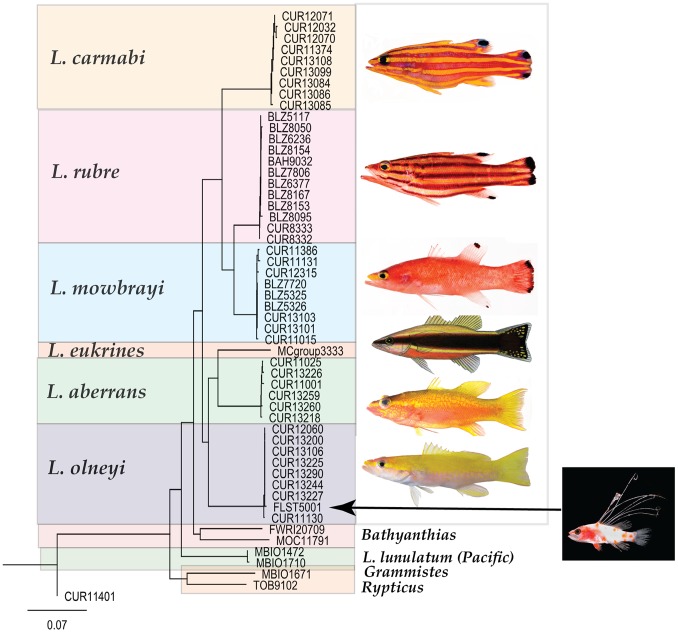
Neighbor-joining tree derived from COI sequences for western Atlantic *Liopropoma* and related taxa. The tree was rooted on *Scorpaena plumieri,* DNA #CUR11401. Divergence represented by scale bar = 7%. Photograph of *Liopropoma rubre* by Carole Baldwin and Lee Weigt; illustration of *L. eukrines* by Raymond Simpson; photograph of larva of *Liopropoma olneyi,* sp. nov., by Cedric Guigand; all other photographs by Ross Robertson and Carole Baldwin.

The tribe Liopropomini comprises the genera *Bathyanthias* Günther, 1880, *Liopropoma* Gill, 1861, and *Rainfordia* McCulloch, 1923, the last of which is restricted to the Indo-Pacific. The unidentified liopropomin larvae differ from known larvae of both *Liopropoma* and *Bathyanthias* in having seven (Fig. 1A) vs. two elongate dorsal-fin spines (Fig. 1B–D) and IX, 11 vs. VIII–IX, 12–13 (*Liopropoma*) or VIII, 13–15 (*Bathyanthias*) dorsal-fin rays [30], [41], [42]. All western Atlantic species of *Liopropoma*–*L. aberrans* (Poey, 1860), *L. carmabi* (Randall, 1963), *L. eukrines* (Starck & Courtenay, 1962), *L. mowbrayi* Woods & Kanazawa,1951, and *L. rubre* Poey, 1861– are represented in our genetic data set and are included in the neighbor-joining tree (Fig. 3) generated from COI sequences (lengths of COI sequences range from 586 to 655 base pairs, average = 650, standard deviation = 14). The liopropomin larva from the Florida Straits does not genetically match any of them (FLST 5001 in Fig. 3). The larva also does not match either of two *Bathyanthias* species represented in the genetic data set (FWRI 20709 and MOC 11791 in Fig. 3). The voucher specimen for one of those genetic samples tentatively was identified as *B. mexicanus* (Schultz, 1958), and the other presumably represents *B. atlanticus* (Schultz, 1958) or *B. cubensis* (Schultz, 1958). All *Bathyanthias* species, including *B. roseus* Günther, 1880, from Pernambuco, Brazil, have more dorsal-fin rays than the liopropomin larvae.

The genetic data (Fig. 3) convincingly match the Florida Straits larva (FLST 5001) with eight adult specimens of a previously undescribed species from deep reefs off Curaçao, *Liopropoma olneyi* sp. nov. Intraspecific variation in COI between the Florida Straits larva and the Curaçao adults is 0.0–0.3%, which is typical of intraspecific variation in other western Atlantic *Liopropoma* species (0.0–0.6%) and well below interspecific values for the group (5.3–15.6%) – Table 1. Corroborating the link between the larva and adults is the unique presence in both of IX, 11 dorsal-fin rays. Below we describe the new species based on eight adult and two larval specimens. The generic assignment of the new species to *Liopropoma* is tentative pending the results of an extensive genetic and morphological study of the Serranidae currently in progress.

**Table 1:** Average (and range) Kimura two-parameter distance summary for species of *Liopropoma* and *Bathyanthias* (and outgroups *Grammistes, Rypticus,* and *Scorpaena*) based on cytochrome c oxidase I (COI) sequences of individuals represented in the neighbor-joining tree in Figure 3.

	*L. aberrans*	*L. carmabi*	*L. eukrines*	*L. lunulatum*
	W. Atl.	W. Atl.	W. Atl.	Indo-Pacific
	(n = 6)	(n = 9)	(n = 1)	(n = 2)
*L. aberrans*	**0.3 (0.0–0.6)**			
*L. carmabi*	14.6 (14.2–15.2)	**0.2 (0.0–0.6)**		
*L. eukrines*	10.5 (10.2–10.8)	15.1 (14.8–15.6)	**na**	
*L. lunulatum*	14.8 (14.6–15.1)	16.4 (16.1–16.9)	14 (14.0–14.1)	**0.1 (0.0–0.2)**
*L. mowbrayi*	12.2 (11.9–12.5)	8.6 (8.2–9.1)	13.5 (13.4–13.9)	15.5 (15.3–15.8)
*L. olneyi*	11.8 (11.5–12.1)	13.6 (13.4–14.2)	13 (12.8–13.1)	14.7 (14.4–14.9)
*L. rubre*	11.9 (11.5–12.4)	10.5 (10.1–10.9)	12.9 (12.8–13.3)	15.8 (15.3–16)
*B. mexicanus*	16.1 (15.8–16.4)	15.2 (14.9–15.8)	16 (−)	16.4 (16.4–16.5)
*Bathyanthias* sp	16.8 (16.5–17)	15.4 (15.2–15.7)	15.4 (−)	16 (15.9–16.1)
*G. sexlineatus*	18.6 (18.3–18.8)	17.9 (17.7–18.4)	18 (−)	17 (16.9–17.1)
*R. carpenteri*	17.3 (17.1–17.5)	17.9 (17.9–18.1)	14.8 (−)	15.4 (15.4–15.5)
*S. plumieri*	21.4 (21.2–21.5)	21.6 (21.4–22)	20.9 (−)	19.8 (19.7–19.8)
	***L. mowbrayi***	***L. olneyi***	***L. rubre***	***B. mexicanus***
	**W. Atl.**	**W. Atl.**	**W. Atl.**	**W. Atl.**
	**(n = 9)**	**(n = 9)**	**(n = 12)**	**(n = 1)**
*L. mowbrayi*	**0.2 (0.9–0.6)**			
*L. olneyi*	13.3 (13.0–13.7)	**0 (0.0–0.3)**		
*L. rubre*	5.7 (5.3–6.0)	12.5 (12.2–13.2)	**0 (0.0–0.3)**	
*B. mexicanus*	13.9 (13.8–14.1)	13.4 (13.4–13.5)	13.8 (13.7–14.3)	**Na**
*Bathyanthias* sp	14.8 (14.6–14.9)	15.2 (15.1–15.4)	14.6 (14.5–14.8)	13.7 (−)
*G. sexlineatus*	18.1 (18.0–18.5)	18.9 (18.7–18.9)	18 (17.8–18.3)	19.8 (−)
*R. carpenteri*	16.8 (16.6–17)	18.6 (18.4–18.6)	17.4 (17.2–17.5)	18.9 (−)
*S. plumieri*	19.7 (19.5–20.4)	20.8 (20.8)	20.3 (20.2–20.6)	19.2 (−)
	***Bathyanthias*** ** sp**	***G. sexlineatus***	***R. carpenteri***	***S. plumieri***
	**W. Atl.**	**Indo–Pacific**	**W. Atl.**	**W. Atl.**
	**(n = 1)**	**(n = 1)**	**(n = 1)**	**(n = 1)**
*Bathyanthias* sp	**na**			
*G. sexlineatus*	15.9 (−)	**na**		
*R. carpenteri*	16.8 (−)	13.2 (−)	**na**	
*S. plumieri*	20.7 (−)	19.5 (−)	19.6 (−)	**na**

Intraspecific averages are shown in bold. “na” = not applicable (n = 1).

***Liopropoma olneyi*** **Baldwin & Johnson, sp. nov.** urn:lsid:zoobank.org:pub:23209F5C-7FFF-4E3D-ACDE-9A03E90025C2.

### 

#### Holotype

USNM 426805, DNA #CUR 13200, GenBank Accession Number (GB) KF770874, 84.2 mm SL, Curaçao, near 12.0832 N, 68.8991W, west of Substation Curaçao downline, Sta. *Curasub* 13–10, 154 m, quinaldine, 5 Aug. 2013, C. Baldwin, R. Robertson, B. Brandt, C. Castillo.

#### Paratypes (Adults)

USNM 406130, DNA #CUR 11130, GB KF770856, 76.0 mm SL, Curaçao, near 12.0832 N, 68.8991 W, Sta. *Curasub* 11-01, 133 m, quinaldine, 23 May 2011, R. Robertson, M. van der Huls, L. Weigt, C. Castillo; USNM 414828, DNA #CUR 12060, GB KF770862, 53.2 mm SL, Curaçao, near 12.0832 N, 68.8991 W, Sta. *Curasub* 12-11, 158–220 m, quinaldine, 6 Aug 2012, C. Baldwin, A. Schrier, B. Brandt, A. Driskell; USNM 426808, DNA #CUR 13225, GB KF770876, 71.5 mm SL, Curaçao, near 12.0832 N, 68.8991 W, off Substation Curacao downline, Sta. CUR13-12, 137 m, quinaldine, 7 Aug 2013, C. Baldwin, R. Robertson, B. van Bebber, C. Castillo; USNM 426809, DNA #CUR 13227, GB KF770878, 67.8 mm SL, Curaçao, near 12.0832 N, 68.8991 W, off Substation Curacao downline, Sta. CUR13-12, 123–171 m, quinaldine, 7 Aug 2013, C. Baldwin, R. Robertson, B. van Bebber, C. Castillo; USNM 426810, DNA #CUR 13244, GB KF770879, 72.2 mm SL, Curaçao, near 12.0832 N, 68.8991 W, east of Substation Curacao downline, Sta. CUR13-13, 183 m, quinaldine, 8 Aug 2013, C. Baldwin, R. Robertson, B. Brandt, C. Castillo, L. Rocha; USNM 426815, DNA #CUR 13290, GB KF770882, 74.8 mm SL, Curaçao, near 12.0832 N, 68.8991 W, off Substation Curacao downline, Sta. CUR13-21, 193 m, quinaldine,17 Aug 2013, C. Baldwin, A. Schrier, B. Brandt, A. Driskell; USNM 422698, DNA #CUR13106, GB KF770872, 73.6 mm SL, Curaçao, near 12.0832 N, 68.8991 W, off Substation Curacao downline, 2013, Substation Curacao, no other data.

#### Paratypes (Postflexion Larvae)

USNM 426868, DNA #FLST 5001, GB KF770883, 14.0 mm SL (cleared and stained), Florida Straits, 24.17016 N, 79.82216 W, Cruise WS-05–15, Sta. M30-013, 25–50 m, MOCNESS 4 M2 mesh size 800 µm, 21 Jun 2005, R. Cowen, C. Guigand; VIMS 13590, 14.7 mm SL, Gulf of Mexico, 27.0000 N, 91.0000 W, 20 Jun 2011, T. Sutton. NOTE: all type material except USNM 426868 was fixed in 10% formalin and preserved in 75% ethanol; USNM 426868 was preserved in 95% ethanol and then cleared and stained.

### Diagnosis

A liopropomin serranid with the following combination of adult and larval characters: dorsal fin IX,11; anal fin III, 8; pectoral fin 14–15; lateral-line scales 46–48 in adults; length of first dorsal spine in adults 4.5% SL; margin of spinous dorsal fin shallowly indented posteriorly in adults (fourth spine 9.7–12% SL, fifth spine only slightly shorter than fourth–8.3–9.3% SL, and sixth – ninth spines approximately subequal–5.5–8.9% SL); second – eighth spines elongate in larvae, 57.1–150% SL; body slender, depth at dorsal-fin origin in adults 23% SL; least caudal-peduncle depth in adults 13–15% SL. Predominant color of adults in life yellow, with yellow covering dorsal portion of trunk, in stripe from tip of upper jaw to posterior edge of operculum, in narrower broken stripe from ventral edge of orbit to posterior edge of operculum, on dorsal and caudal fins, and in stripe on anal fin; top of head dark rose/orange, ventral portions of head and trunk pale rose to white; dorsal and ventral portions of trunk with scattered yellow spots. In alcohol, head and trunk uniformly pale, slightly paler on abdomen and throat. Larvae with conspicuous pattern of orange chromatophores in life on head and trunk, orange pigment on elongate second dorsal-fin spine associated with swellings in sheath covering spine, several orange blotches on anal-fin rays, large blotch of orange pigment covering most of pelvic fin, and melanophores on spinous dorsal fin (see description for full characterization of pigment in larvae). Note: in the absence of information on pigmentation in larvae of most western Atlantic *Liopropoma,* the diagnostic aspects of pigment in larval *L. olneyi* are unknown; however, non-melanistic color patterns in marine teleost larvae are typically species specific [19]).

### Description of Adults

**Figure 4:**
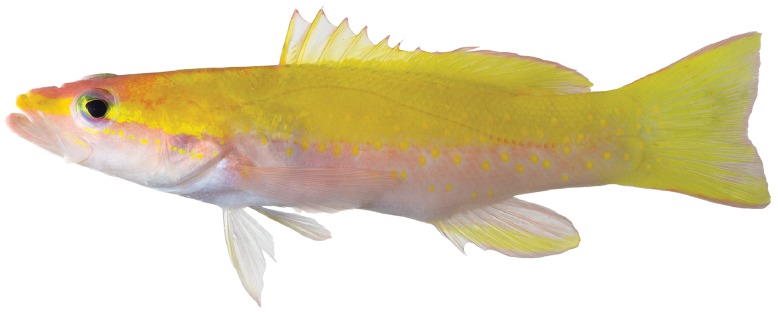
*Liopropoma olneyi,* sp. nov., USNM 426805, holotype, DNA #CUR 13200, 84.3 mm SL. Photo by Ross Robertson and Carole Baldwin.

(Fig. 4). Counts and measurements of holotype are given in Table 2. Dorsal-fin rays IX, 11 (one specimen, USNM 414828, with an aberrant dorsal-fin configuration such that externally the specimen has 11 soft rays but internally a tiny additional ray and pterygiophore are present as the ultimate element); anal-fin rays III, 8; pectoral-fin rays 14 or 15; pelvic-fin rays I, 5; principal caudal-fin rays 17, branched caudal-fin rays 15, procurrent caudal-fin rays 17–18; pored lateral-line scales 46–48; gillrakers on first arch, including rudiments, 5–6+12–13, total 17–19; vertebrae 10+14 = 24.

**Table 2:** Selected counts and measurements for holotype of *Liopropoma olneyi* sp. nov., and *Liopropoma aberrans* from Robins [8]. Measurements are in percentages of SL.

	*Liopropoma olneyi*	*Liopropoma aberrans*
	USNM 426805	UMML 22324
SL	84.3 mm SL	112 mm SL
Dorsal Fin	IX, 11	VIII, 12
Anal Fin	III, 8	III, 8
Pectoral Fin	14, 15	14, 14
Lateral-line scales	48	47
Gill Rakers	5+12 = 17	14*
Head length	37	40
Snout length	8.8	9.8
Orbit diameter	8.1	7.2
Bony interorbital	5.9	6.4
Body depth at dorsal-fin origin	23	32
Least depth of caudal peduncle	15	14**
Pectoral-fin length	27	28
Pelvic-fin length	18	22
Lengths of dorsal-fin spines:		
I	4.5	2.8
II	11	10
III	12	12
IV	10	9.4
V	9.0	4.0
VI	8.6	4.0
VII	7.5	4.0
VIII	6.5	4.5
IX	6.4	–

*Based on Robins’ [8∶592] description of gill rakers, he did not count the rudimentary pads on the lower limb of the first arch, only those on the upper limb.

**Robins [8∶592] erroneously gave this value as 7.0. The least depth of caudal peduncle was re-measured by R. Robins (pers. comm., 2 Oct 2013) as 15.2 mm, or 14% SL.

Body proportions expressed as % of SL: body depth at origin of dorsal fin 20–24; body width just behind gill opening 13–14; head length 35–39; snout length 8.0–9.6; orbit diameter 8.1–9.4; least distance between nostrils 2.2–5.5; bony interorbital width 4.7–5.9; upper-jaw length 14–16; greatest depth of maxilla 4.8–5.4; least caudal-peduncle depth 13–15; caudal-peduncle length19–22; lengths of dorsal-fin spines: (I) 3.4–4.5, (II) 10–12, (III) 11–13, (IV) 10–12, (V) 8.3–9.3, (VI) 7.3–8.9, (VII) 5.6–7.0, (VIII) 5.5–7.0, (IX) 5.4–7.7; longest dorsal soft ray the 8^th^, length 17–21; length of 3^rd^ anal-fin spine 9.2–12; longest anal soft ray the 4^th^ or 5^th^, length 18–20; caudal-fin length 22–28; pectoral-fin length 19–27, fin reaching vertical through base of 9^th^ dorsal spine; pelvic-fin length 16–20, fin reaching vertical through base of 6^th^ dorsal spine.

Interorbital region flat; mouth oblique, maxilla reaching vertical short of posterior edge of pupil; prominent bony projection on posteroventral corner of maxilla; lower jaw slightly projecting. Anterior nostril in thin membranous tube, nostril situated at edge of groove between front of snout and upper lip; posterior nostril with short flap on anterior margin, nostril situated at point about two widths of nostril anterior to orbit. Lateral line strongly arched above pectoral fin, highest point below sixth dorsal-fin spine; about 3 rows of scales between lateral line and base of this spine; lateral line continuing onto base of caudal fin.

Trunk covered with ctenoid scales, scales becoming weakly ctenoid anteriorly and cycloid on head. Head fully scaled except lips, gular region, and branchiostegals naked. Some small, elongate scales along base of spinous dorsal fin, scales extending further distally on posterior portion of fin; soft dorsal fin with scales along base, fewer scales present posteriorly. Each anal- fin element with column of three small elongate scales at base, number of scales in each column reducing to two posteriorly; membrane between third anal-fin spine and first soft ray with scales extending almost to distal edge; membranes of anterior anal soft rays with scales on at least proximal two-thirds, and membranes of posterior soft rays with scales basally; no scales on membranes of first through third spines. Scales present on pectoral-fin base, and elongate scales present on proximal portion of fin. No scales on pelvic fin; pelvic axillary scales present. Caudal fin almost completely covered with scales, scales becoming smaller and more elongate distally.

Jaw teeth small and depressible; upper and lower jaws with bands of villiform teeth, bands broadest anteriorly; largest teeth present along posterior portion of lower jaw. Vomer with chevron-shaped patch of small teeth. Palatine with teeth in long narrow band. Three flattened spines on opercle, middle spine most robust, largest, and exposed; other two spines less exposed. Margin of preopercle with small serrations on upper limb, largest serrations on ventral portion of upper limb; no serrations on lower limb.

Prior to preservation, dorsal fin yellow, with thin, pinkish-rose stripe along distal margin, stripe slightly thicker on soft dorsal fin; a bit of additional pinkish rose pigment at base and on membrane of last dorsal soft ray. Caudal fin yellow, with orange to pinkish-rose stripe along dorsal and ventral margins; pinkish tinge at distal tips of central rays, sometimes extending anteriorly as streaks of various length on one or more rays of ventral lobe. Anal fin pale rose with a broad yellow stripe extending from bases of anal-fin spines posteroventrally to distal tip of fin. Pelvic fin clear except yellow present on and between spine and first one or two soft rays; in some specimens, base of pelvic fin at junction with abdomen rosy. Pectoral fin pale rose, a small blotch of yellow pigment present on bases of uppermost rays; sometimes a patch of rose pigment present posterior to this blotch that is darker than background fin color.

A broad, longitudinal swath of dark rose/orange pigment extending from anterior tip of maxilla to origin of dorsal fin, often narrowing posteriorly to thin stripe immediately in front of dorsal fin; this swath often the darkest pigment on body; behind posterodorsal portion of orbit, this rose/orange swath sometimes uniform and sometimes divided into dorsal and ventral portions by yellow pigment encroaching anteriorly from trunk; when uniform, swath extending posteriorly as faint orange spots on dorsal portion of trunk. Yellow stripe extending from anterior tip of premaxilla through eye to posterior edge of operculum or slightly beyond; second yellow stripe extending from along ventral margin of orbit posteriorly (or slightly posteroventrally) to posterior edge of operculum; this stripe variously breaking into spots or blotches as far forward as orbit; dorsal and ventral portions of iris typically rose; tip of lower jaw rose; dorsal margin of maxilla usually with small yellow stripe posteriorly; remainder of head pale rose to white. Dorsal portion of trunk yellow, fading to rose near midbody and white on abdomen; scattered yellow spots present on trunk and extending onto anterior portion of caudal fin; spots usually extending deeper into ventral portion of trunk below middle of spinous dorsal fin; yellow spots evident even against yellow background color of dorsal portion of trunk. Section of posterior portion of lateral line with dark rose to orange crescent-shaped marking on each scale; this pigment not evident on smallest adult examined. In alcohol, head and trunk uniformly pale, slightly paler on abdomen and throat.

### Description of Larvae

**Figure 5:**
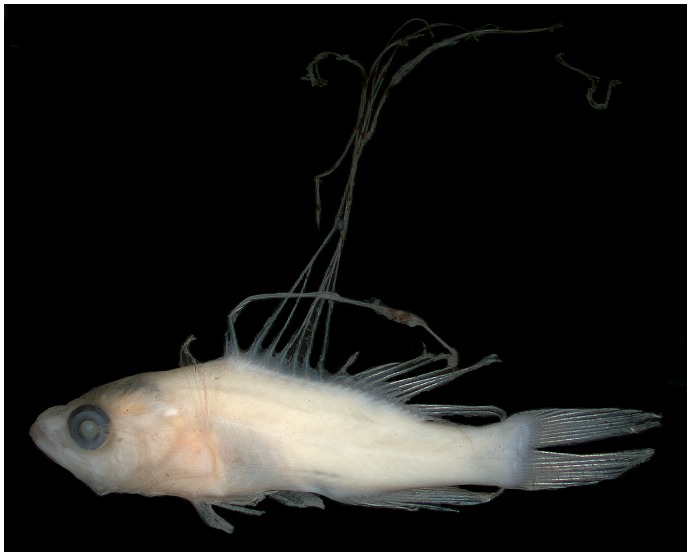
*Liopropoma olneyi,* sp. nov., VIMS 13590, paratype, 14.7 mm SL. Photo by G. David Johnson.

**Figure 6:**
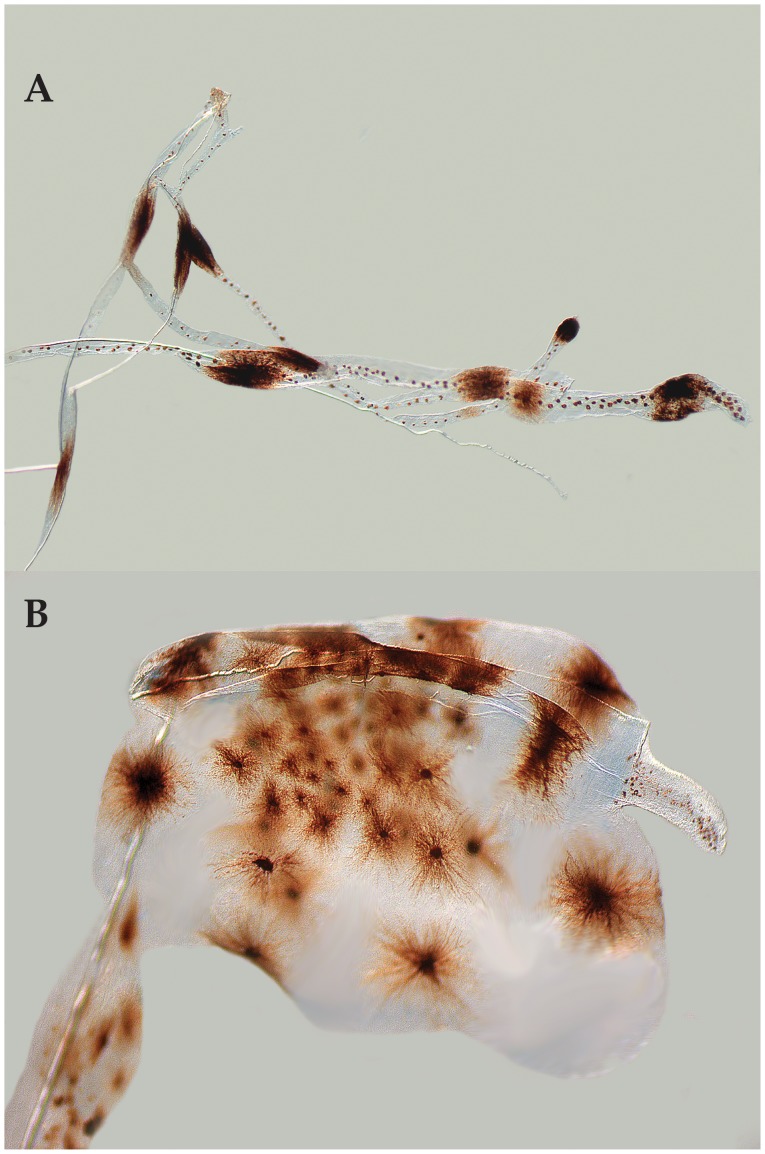
Close-up images of swellings and pigmentation on elongate dorsal-fin spines in cleared larval *Liopropoma olneyi,* sp. nov., USNM 426868, paratype, 14.0 mm SL. A, portions of several of the third through eighth dorsal-fin spines. B, distal end of second dorsal-fin spine. Photos by G. David Johnson.

(Figs. 1A, 2, 5, 6) Counts and measurements of USNM 426868 are given first if different from those of VIMS 13590. Some significant differences are explained by the different preparations of the two specimens (fixation in 95% ethanol vs. 10% formalin). Counts of dorsal-, anal-, pelvic fins and vertebrae same as those given for adults; pectoral-fin rays 14. Body laterally compressed, moderately deep (depth at pectoral-fin base 31 and 29% SL, maximum depth at caudal peduncle 15 and 13% SL); head large, 36 and 41% SL; anus located just posterior to mid body, preanal length 59% SL; eye round and moderately large, greater in diameter than length of snout in USNM specimen (eye diameter 10% SL vs. snout length 7.1% SL), smaller in diameter than length of snout in VIMS specimen (eye diameter 8.8% SL vs. snout length 10% SL); mouth large, maxilla nearly reaching vertical through middle of eye; pectoral fin broken in USNM specimen, longest ray of pectoral fin in VIMS specimen 20% SL; longest ray in pelvic fin 23% SL, fin damaged in VIMS specimen; dorsal-fin spines ranging in length from 3.6% SL (first spine in USNM larva) to 150% SL (third spine in VIMS larva); first and ninth spines smallest (3.6–5.7% SL); second through eighth spines elongate (72–126% SL and 57–150% SL), third spine longest (126 and 150% SL); first anal-fin spine shortest (5.7% SL in USNM specimen), second and third spines nearly equal (10 and 11% SL, respectively, in USNM specimen – second spine broken in VIMS specimen and spines not measured). Elongate dorsal-fin spines thin and flexible; sheath of second spine with several swellings, distalmost of these greatly enlarged in USNM specimen (presumably broken in VIMS specimen); sheaths of remaining elongate spines with no or only slight swellings. Preopercle with six small spines on posterior margin (medial ridge), three tiny spines on lateral ridge; interopercle and subopercle each with one small spine; spines on opercle as in adults; supraorbital ridge with three tiny spines. Several small scales present on opercle and preopercle. Frontals without rugosity.

Pigment in preservative comprising several melanophores on frontals, a cap of internal melanophores over swimbladder, melanophores associated with posterior swellings of second dorsal-fin spine (large bulb at tip of spine in USNM specimen with numerous conspicuous stellate melanophores – Fig. 6B), four to eight small blotches of melanophores along length of each of third through eighth dorsal spines, and tiny melanophores along length of each of those spines between blotches (Fig. 6A). In addition to this pigment, in life larvae with multiple patches of orange-red pigment: most of head covered with orange-red pigment that extends posteriorly onto pectoral-fin base and anterior portion of trunk; top of head from mid orbit to near tip of snout white, lower jaw mostly white, nape from vertical through angle of preopercle to origin of dorsal fin white, and thoracic region white. Most of trunk white except for the following: four roughly circular blotches of orange pigment present along dorsal portion of trunk and caudal peduncle; an additional faint blotch present between the anteriormost two; four roughly circular blotches of orange pigment present along ventral portion of trunk from abdomen to caudal peduncle; dorsal and ventral trunk blotches arranged roughly linearly; two large orange blotches present mid-laterally on caudal peduncle between dorsal and ventral series; oblong patch of orange pigment present at bases of dorsal spines III–V; other oblong patches of orange pigment present at bases of dorsal-fin soft rays 4–7 (bleeding onto upper body) and anal-fin soft rays 2–5; approximately five small blotches of orange pigment present along elongate second dorsal-fin spine associated with swellings of sheath covering spine; several orange blotches present on anal-fin rays; a large blotch of orange pigment covering most of pelvic fin; basal portion of pectoral fin appearing orange, but unable to determine if this pigment located on or beneath fin.

### Etymology

In remembrance of John E. Olney–beloved friend, colleague, and teacher.

#### Common name

Yellow-spotted golden bass, in reference to the yellow spots on the dorsal and ventral portions of the trunk in adults. Along with other characters, these spots distinguish *L. olneyi* from the other western Atlantic “golden bass” of the genus *Liopropoma*, *L. aberrans.*

### Distribution

Adults are known from images and specimens from Curaçao, southern Caribbean. Larvae are known from the Florida Straits and Gulf of Mexico.

### Habitat

**Figure 7:**
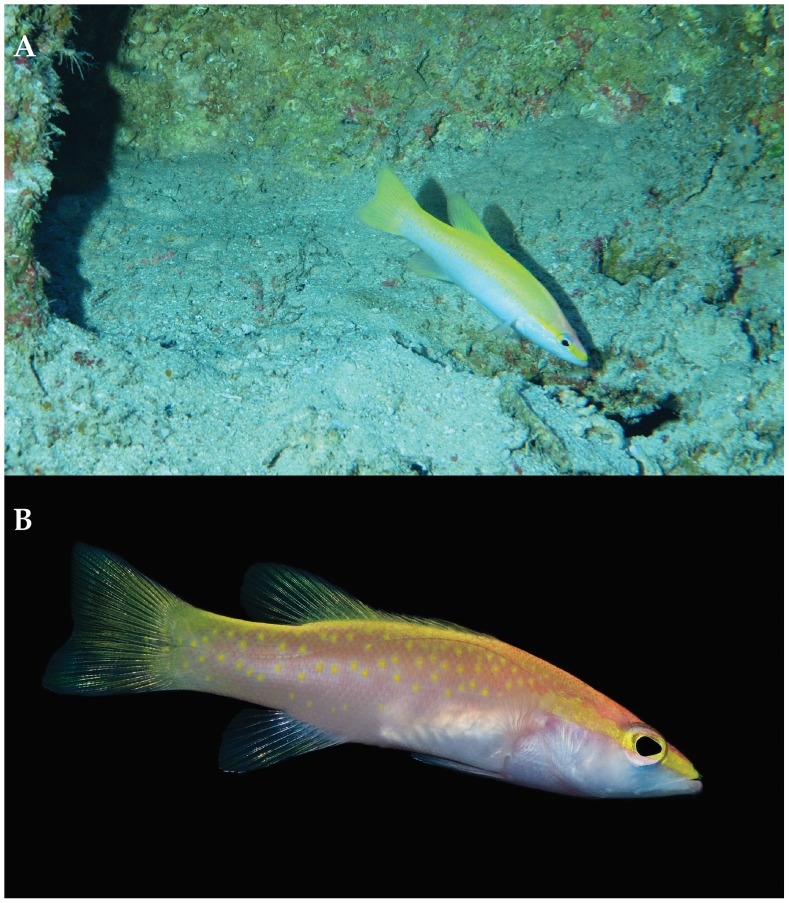
*Liopropoma olneyi,* sp. nov. A, in-situ photograph taken from the *Curasub* submersible at 125 m on the reef slope adjacent to the Curacao Sea Aquarium on 7 Aug 2013. Photo courtesy of Substation Curacao. B, aquarium photograph by Barry Brown.

Off Curaçao, *Liopropoma olneyi* is found from 123–220 m inhabiting rocky slopes and ledges. It retreats into small caves and crevices when threatened. Figure 7A shows an in-situ photograph taken from the *Curasub* submersible at 125 m on the reef slope adjacent to the Curacao Sea Aquarium. The larva from Florida Straits was captured between 25 and 50 m.

### Remarks

**Figure 8:**
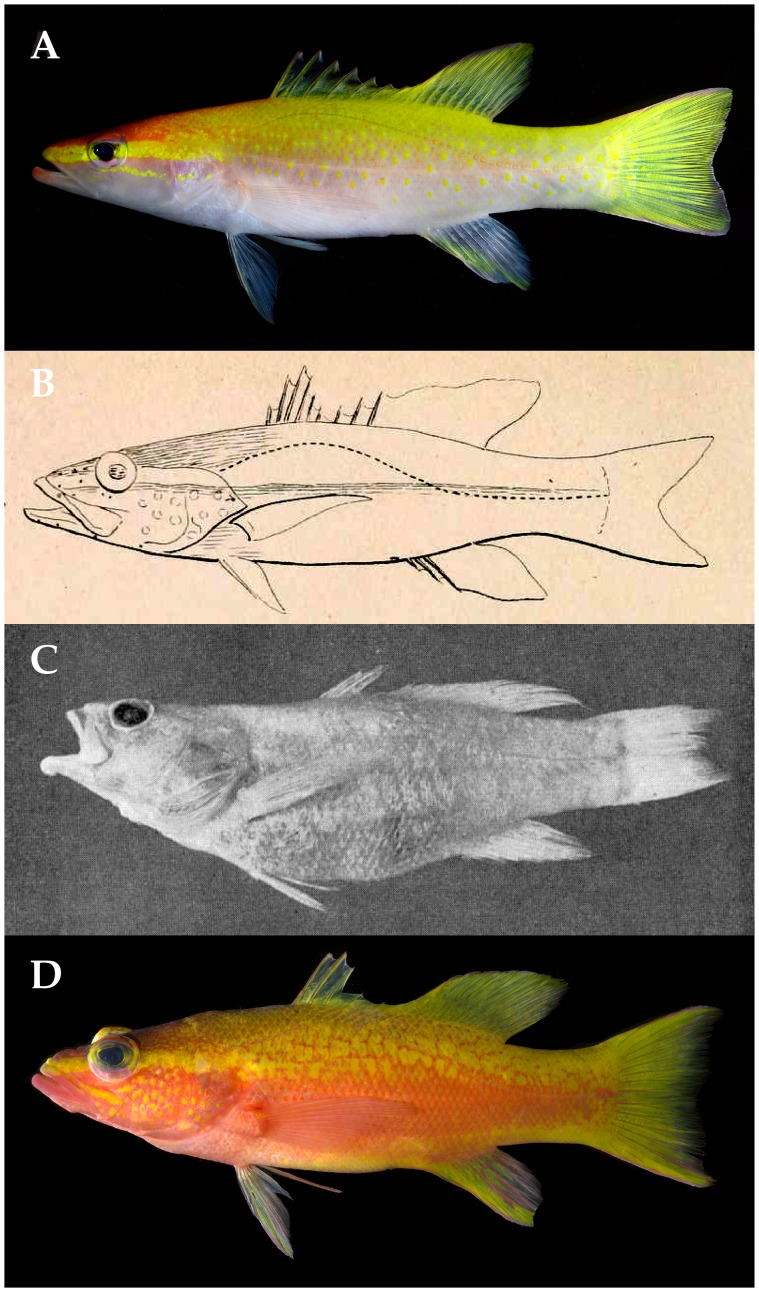
Comparison of *Liopropoma olneyi,* sp. nov., and *L. aberrans.* A, *L. olneyi*, Curacao (USNM 426808, paratype, DNA #CUR12225, 73 mm SL). B, *L. aberrans,* Cuba (illustration from original description [21: Plate 12–2], 115 mm SL). C, *L. aberrans,* Bahamas (photograph from redescription of *L. aberrans* [6: Fig. 1], UMML 22324, 112 mm SL). D, *L. aberrans,* Curacao (USNM 426807, DNA #CUR 12226, 102 mm SL). Photos A and D by Ross Robertson and Carole Baldwin.

Poey [43] described *Perca aberrans* (now *Liopropoma aberrans –* [32], [43] from a single specimen collected off Cuba that he reported had nine dorsal-fin spines (the whereabouts of the holotype are unknown [44]). Until the discovery of *L. olneyi,* the presence of nine dorsal spines was unique among western Atlantic liopropomins to *L. aberrans,* which initially suggested to us that our Curaçao species might be Poey’s *L. aberrans.* (Note: western Atlantic *Bathyanthias roseus* was erroneously characterized as having nine dorsal spines [40], but as noted by Baldwin & Johnson [27], the holotype has eight.) However, unlike *L. aberrans,* which Poey reported as having 12 dorsal soft rays, *L. olneyi* has 11 (the posterior portion of the soft dorsal fin is aberrant in one specimen, USNM 414828, with 11 external soft rays but a tiny12th pterygiophore). Furthermore, Poey’s illustration of *P. aberrans* ([43], Fig. 8B herein) shows a deep indentation in the spinous dorsal fin resulting from the fifth spine being much shorter than the fourth, and very short sixth and seventh dorsal-fin spines. In *L. olneyi* (Fig. 4, 8A), there is only a shallow indentation in the spinous dorsal fin, with the fifth spine only slightly shorter than the fourth, and the sixth-ninth dorsal spines similar in height and only slightly shorter than the fifth. Poey’s illustration also shows a deeply indented caudal fin, which is not present in *L. olneyi.*

Color patterns also are different between the two species. In the original description of *L. aberrans* [43], Poey described the head as rose and the rest of the body as orange. In *L. olneyi*, the trunk is mostly yellow dorsally and pale rose to white ventrally. Poey stated that the pink of the head extends onto the throat, but in *L. olneyi* the throat is almost white. He described round orange spots on the cheek and depicted spots in his illustration (Fig. 8B) as scattered on the cheek; *L. olneyi* lacks scattered spots on the cheek but usually has a series of yellow spots forming a stripe that extends from beneath the orbit posteriorly to the edge of the opercle. The only yellow pigment that Poey mentioned for *P. aberrans* is on the iris, but yellow is the predominant color on the body and fins of *L. olneyi*. In his illustration, Poey depicted a stripe through the eye similar to the yellow one in the new species, but he did not mention this stripe in his description. Poey stated that the dorsal, caudal, and anal fins are orange, except that the anterior portion of the anal fin is pink. In *L. olneyi*, the dorsal and caudal fins are largely yellow, and the anal fin has a yellow stripe on a pale rose background. Even if yellow and orange are confused in his description, the presence of scattered spots on the cheek in Poey’s illustration and their absence in the new species is sufficient evidence that *L. olneyi* is not Poey’s *L. aberrans.*

*Liopropoma aberrans* was redescribed by Robins [30] on the basis of one specimen caught off the Bahamas (Fig. 8C). Robins concluded that his specimen agrees well with the original description, in particular with regards to the color pattern. However, we note some differences based on the two descriptions. A general description of the color of the head was not provided for the Bahamas specimen, but a bright yellow stripe extending from the snout to the edge of the opercle was noted, as were yellow dorsal, anal, and caudal fins and yellow spots on the upper part of the body [30]. As noted above, the only mention of yellow pigment in the original description of *L. aberrans* [43] was on the iris. Furthermore, Robins [30] did not mention spots on the cheek as illustrated by Poey (Fig. 8B). Unfortunately, the color slide upon which his description was based is missing. Robins [30] noted that his specimen has eight dorsal spines whereas Poey’s has nine, but he attributed this to unusual variation or an incorrect original determination. We note that the original illustration of *L. aberrans* (Fig. 8B) clearly shows nine dorsal spines. There is thus the possibility that Robins’ specimen of *L. aberrans* (Fig. 8C) represents a different species. Location of the holotype of Poey’s *L. aberrans* or collection of a specimen from the type locality (Cuba) with the same fin-rays counts and color pattern would help resolve the status of Robins’ *L. aberrans.*

*Liopropoma olneyi* (Figs. 4, 8A) is distinct from Robins’ [30] *L. aberrans.* Robins described the upper part of the body has having yellow spots on a rosy to orangish background, the spots changing to orange below the lateral line. In *L. olneyi,* the upper part of the body has yellow spots on a yellow background, and the spots remain yellow below the lateral line. Additionally (Table 2), *L. olneyi* is more slender than Robins’ *L. aberrans,* has a longer first dorsal-fin spine, and lacks a deep indentation at the posterior end of the spinous dorsal fin (i.e., has longer fifth-eighth dorsal-fin spines).

Robins’ [30] *L. aberrans* (Fig. 8C) better matches another series of *Liopropoma* specimens collected by submersible off Curaçao and referred to here as “Curacao *L. aberrans*” (Fig. 8D). Both have a yellow stripe on the head from the snout to the edge of the operculum, and both have yellow spots on a rosy to orangish background on the dorsal portion of the trunk. Robins indicated that these spots occur on each scale, but in Curaçao *L. aberrans*, most of the spots are larger and cover more than one scale. As noted, Robins’ stated that the yellow spots change to orange below the lateral line; in Curaçao *L. aberrans,* the trunk beneath the lateral line is orange with yellow blotches until approximately mid body, orange at mid body, and with a yellow stripe ventrally that extends from the abdomen to the caudal fin. Robins did not mention this yellow stripe. Robins described the dorsal, anal, and caudal fins as yellow; in Curaçao *L. aberrans*, those fins are mostly yellow, but the caudal and anal fins have prominent orange streaks. Counts of Robins’ specimen match those of Curaçao *L. aberrans*, especially the presence of VIII, 12 dorsal-fin rays and 14 pectoral-fin rays. The spinous dorsal fin is deeply indented posteriorly in Curaçao *L. aberrans*, Robins’ *L. aberrans*, and Poey’s *L. aberrans,* and we tentatively recognize all of them as *L. aberrans* pending further study. We note that a color photograph of a specimen of *L. aberrans* from Jamaica (Patrick Colin, pers. comm.) shows a color pattern nearly identical to that of Curaçao *L. aberrans.* That color pattern (Fig. 8D) is distinct from the color patterns of *L. olneyi* (Figs. 4, 7, 8A). In *L. olneyi* the dorsal half of the trunk is yellow with distinctive yellow, round spots, whereas in Curaçao *L. aberrans*, the dorsal half of the trunk is orange with yellow spots or larger, irregular yellow blotches. The ventral portion of the trunk in *L. olneyi* is pale rose fading to white on the abdomen, and the ventral portion of the head also is white. In Curaçao *L. aberrans*, the ventral portion of the trunk is orange changing to yellow, and the ventral portion of the head is conspicuously rose to orange. In *L. olneyi*, there are no produced caudal-fin rays, whereas in the Curaçao *L. aberrans* the outer tips of the upper and lower caudal lobes are slightly produced. Finally, *L. olneyi* has yellow spots in a series that form a stripe from the lower portion of the orbit to the edge of the opercle; in Curaçao *L. aberrans*, there is no yellow stripe from the lower portion of the orbit to the edge of the opercle, and there are scattered yellow spots on the cheek and opercle. Similar cheek spots are conspicuously illustrated in Poey’s original description of *L. aberrans* (Fig. 8B). We further note that *L. olneyi* and Curaçao *L. aberrans* are well separated by an average of 12% genetic distance in COI, a value consistent with interspecific divergence within *Liopropoma* (Table 1).

**Figure 9:**
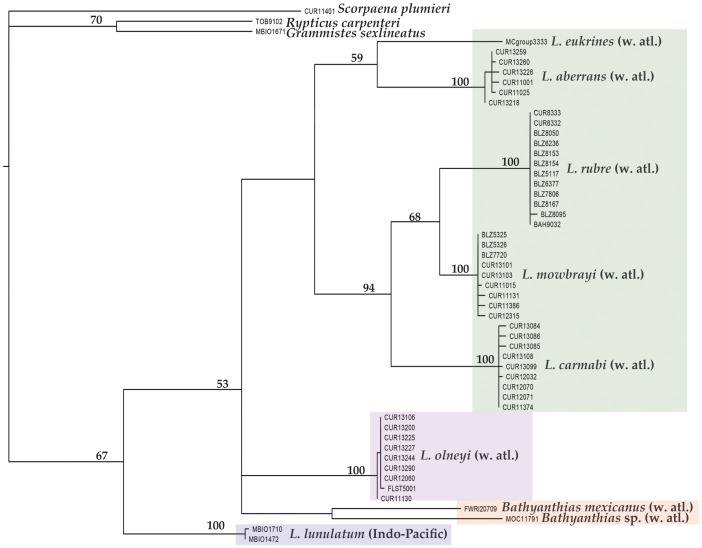
The strict consensus tree of a maximum parsimony analysis of the COI region among western Atlantic *Liopropoma* and related taxa. Numbers above branches represent bootstrap support values.

*Liopropoma olneyi* is easily distinguished from *L. carmabi, L. eukrines, L. mowbrayi,* and *L. rubre* by color pattern (Fig. 3) and from those species and the related *Bathyanthias* (*B. atlanticus, B. cubensis, B. mexicanus, B. roseus*) by numbers of dorsal-fin rays (IX, 11 in *L. olneyi;* VIII, 12–13 in the other *Liopropoma;* VIII, 13–15 in *Bathyanthias*). A preliminary phylogeny of this group based on maximum parsimony analysis of the COI sequences (Fig. 9) suggests that the generic classification of liopropomins may require revision. An Indo-Pacific species of *Liopropoma, L. lunulatum,* falls outside of the western Atlantic *Liopropoma*+*Bathyanthias* clade, and *Bathyanthias* and *L. olneyi* form an unresolved trichotomy with the remaining western Atlantic *Liopropoma.* Addition of the Cape Verdes *L. emanueli* Wirtz & Schliewen 2012, eastern Pacific *L. longilepis* Garman 1899, and more of the Indo-Pacific species to future phylogenetic analyses of the group is planned.

### Comparative Material Examined

Specimens, color images, or both, were examined of all material listed in Appendix S1.

## Conclusions

*Liopropoma olneyi* is the only liopropomin serranid known to have a pelagic larval stage with seven elongate dorsal-fin spines; all other known liopropomins have only two (Fig. 1, [19], [24], [25]). This feature, combined with the presence of IX dorsal-fin spines (unique among liopropomins except for Poey’s [43] single specimen of *L. aberrans*), could indicate that *L. olneyi* warrants a new generic assignment, and further study of generic relationships among liopropomin serranids is in progress. Knowledge of larval morphology for all liopropomin species may shed light on generic relationships, but other than *L. olneyi,* species identifications of larvae exist only for *L. carmabi* and *L. rubre* (from aquarium-reared specimens).

This study demonstrates that enhancing genetic databases of marine fishes through exploratory sampling in underexplored ecosystems such as deep reefs improves our ability to identify larvae. It further shows that genetic databases, if sufficiently populated, enable detection of putative new species in underexplored ecosystems such as Caribbean deep reefs. The Smithsonian DNA barcoding database includes adults of all known species of western Atlantic *Liopropoma,* which enabled detection of *L. olneyi,* but depths of 50–300 m circumtropically are so poorly explored that genetic databases for most taxa are undoubtedly incomplete. Continued exploration of deep reefs to improve genetic databases is needed. Combined with traditional morphological investigation and comparative analysis of fresh color patterns, genetic data are proving valuable in elucidating species diversity of the Caribbean fish fauna.

## Supporting Information

Appendix S1 **Links between DNA voucher specimens, GenBank accession numbers, and cytochrome c oxidase subunit I (COI) sequences of** ***Liopropoma olneyi*** **sp. nov., related Liopropomini, and outgroup taxa.**
(DOCX)
