# Supplementary material for: Connectivity across the Caribbean Sea: DNA Barcoding and Morphology Unite an Enigmatic Fish Larva from the Florida Straits with a New Species of Sea Bass from Deep Reefs off Curaçao
Source: PLoS One. 2014 May 13;9(5):e97661. doi: 10.1371/journal.pone.0097661 (PMC4019605; doi:10.1371/journal.pone.0097661)
Supplement: Appendix S1 — Links between DNA voucher specimens, GenBank accession numbers, and cytochrome c oxidase subunit I (COI) sequences of Liopropoma olneyi sp. nov., related Liopropomini, and outgroup taxa. (DOCX) [file pone.0097661.s001.docx]

**Appendix S1. Links between DNA voucher specimens, GenBank accession numbers, and cytochrome c oxidase subunit I (COI) sequences of *Liopropoma olneyi* sp. nov., related Liopropomini, and outgroup taxa.**

| Catalog Number/DNA Number | GenBank No. | GenSeq Designation |
| --- | --- | --- |
| *Liopropoma olneyi* |  |  |
| USNM 426805, CUR 13200, Holotype | KF770874 | genseq-1 COI |
| USNM 406130, CUR 11130, Paratype | KF770856 | genseq-2 COI |
| USNM 414828, CUR 12060, Paratype | KF770862 | genseq-2 COI |
| USNM 426808, CUR 13225, Paratype | KF770876 | genseq-2 COI |
| USNM 426809, CUR 13227, Paratype | KF770878 | genseq-2 COI |
| USNM 426810, CUR 13244, Paratype | KF770879 | genseq-2 COI |
| USNM 426815, CUR 13290, Paratype | KF770882 | genseq-2 COI |
| USNM 422698, CUR13106, Paratype | KF770872 | genseq-2 COI |
| USNM 426868, FLST 5001, Paratype (larva) | KF770883 |  |
| *Liopropoma aberrans* |  |  |
| USNM 406001, CUR 11001 | KF770853 | genseq-4 COI |
| USNM 406025, CUR 11025 | KF770855 | genseq-4 COI |
| USNM 426806, CUR 13218 | KF770875 | genseq-4 COI |
| USNM 426807, CUR 13226 | KF770877 | genseq-4 COI |
| USNM 426814, CUR 13259 | KF770880 | genseq-4 COI |
| USNM 426812, CUR 13260 | KF770881 | genseq-4 COI |
| *Liopropoma carmabi* |  |  |
| USNM 406374, CUR 11374 | KF770858 | genseq-4 COI |
| USNM 414825, CUR 12032 | KF770861 | genseq-4 COI |
| USNM 414826, CUR 12070 | KF770863 | genseq-4 COI |
| USNM 414827, CUR 12071 | KF770864 | genseq-4 COI |
| USNM 413959, CUR 13084 | KF770866 | genseq-4 COI |
| USNM 413960, CUR 13085 | KF770867 | genseq-4 COI |
| USNM 413961, CUR 13086 | KF770868 | genseq-4 COI |
| USNM 422694, CUR 13099 | KF770869 | genseq-4 COI |
| USNM 422687, CUR 13108 | KF770873 | genseq-4 COI |
| *Liopropoma eukrines* |  |  |
| SIO 01-11, MCgroup 3333 | KF770885 | genseq-4 COI |
| *Liopropoma mowbrayi* |  |  |
| USNM 420350, BLZ 5325 | JQ840569 | genseq-4 COI |
| USNM 420349, BLZ 5326 | JQ840570 | genseq-4 COI |
| BLZ 7720 (photo voucher only) | JQ841243 | genseq-5 COI |
| USNM 406015, CUR 11015 | KF770854 | genseq-4 COI |
| USNM 406131, CUR 11131 | KF770857 | genseq-4 COI |
| USNM 406386, CUR 11386 | KF770859 | genseq-4 COI |
| USNM 414815, CUR 12315 | KF770865 | genseq-4 COI |
| USNM 422684, CUR 13101 | KF770870 | genseq-4 COI |
| USNM 422675, CUR 13103 | KF770871 | genseq-4 COI |
| *Liopropoma rubre* |  |  |
| USNM 414697, BAH 9032 | KF770852 | genseq-4 COI |
| USNM 419340, BLZ 5117 | JQ840571 | genseq-4 COI |
| USNM 416331, BLZ 6236 | JQ840899 | genseq-4 COI |
| USNM 416379, BLZ 6377 | JQ840900 | genseq-4 COI |
| USNM 416009, BLZ 7806 | JQ841244 | genseq-4 COI |
| USNM 415207, BLZ 8050 | JQ841640 | genseq-4 COI |
| USNM 415226, BLZ 8095 | JQ841637 | genseq-4 COI |
| USNM 415180, BLZ 8153 | JQ841638 | genseq-4 COI |
| USNM 415181, BLZ 8154 | JQ841641 | genseq-4 COI |
| USNM 415244, BLZ 8167 | JQ841639 | genseq-4 COI |
| USNM 414498, CUR 8332 | JQ842192 | genseq-4 COI |
| USNM 414499, CUR 8333 | JQ842193 | genseq-4 COI |
| *Liopropoma lunulatum* (Pacific) |  |  |
| MBIO 1710 (no photo or specimen voucher) | JQ431889 | no classification |
| MNHN 2008-1023, MBIO 1472 | JQ431888 | genseq-4 COI |
| *Bathyanthias mexicanus* |  |  |
| FSBC 20709, FWRI 20709 | KF770884 | genseq-4 COI |
| *Bathyanthias* sp. |  |  |
| USNM 407791, MOC 11791 | KF770886 | genseq-4 COI |
| Outgroup Taxa |  |  |
| *Grammistes sexlineatus* |  |  |
| MNHN 2008-1105, MBIO 1671 | JQ431776 | genseq-4 COI |
| *Rypticus carpenteri* |  |  |
| USNM 401296, TOB 9102 | JN828097 | genseq-4 COI |
| *Scorpaena plumieri* |  |  |
| USNM 406401, CUR 11401 | KF770860 | genseq-4 COI |
